# High β-Glucan Barley Supplementation Improves Glucose Tolerance by Increasing GLP-1 Secretion in Diet-Induced Obesity Mice

**DOI:** 10.3390/nu13020527

**Published:** 2021-02-06

**Authors:** Sachina Suzuki, Seiichiro Aoe

**Affiliations:** 1 The Institute of Human Culture Studies, Otsuma Women’s University, Chiyoda-ku, Tokyo 102-8357, Japan; suzusachi0421@gmail.com; 2 Studies in Human Life Sciences, Graduate School of Studies in Human Culture, Otsuma Women’s University, Chiyoda-ku, Tokyo 102-8357, Japan

**Keywords:** barley, β-glucan, L cell, glucagon-like peptide 1 (GLP-1), glucose tolerance

## Abstract

The aim of this study was to investigate the underlying mechanism for the improvement of glucose tolerance following intake of high β-glucan barley (HGB) in terms of intestinal metabolism. C57BL/6J male mice were fed a fatty diet supplemented with HGB corresponding to 5% of dietary fiber for 83 days. An oral glucose tolerance test was performed at the end of the experimental period. The concentration of short-chain fatty acids (SCFAs) in the cecum was analyzed by GC–MS (gas chromatography–mass spectrometry). The mRNA expression levels related to L cell function in the ileum were measured by real-time PCR. Glucagon-like peptide-1 (GLP-1) levels in the portal vein and cecal content were assessed by enzyme-linked immunosorbent assay. GLP-1-producing L cells of the ileum were quantified by immunohistochemistry. HGB intake improved glucose tolerance and increased the cecal levels of SCFAs, acetate, and propionate. The number of GLP-1-positive L cells in the HGB group was significantly higher than in the control group. GLP-1 levels in the portal vein and cecal GLP-1 pool size in the HGB group were significantly higher than the control group. In conclusion, we report improved glucose tolerance after HGB intake induced by an increase in L cell number and subsequent rise in GLP-1 secretion.

## 1. Introduction

A diet that includes barley has several beneficial effects, such as lowering the postprandial glucose rise [1,2,3] and improved blood cholesterol concentration [4,5,6]. Barley is rich in β-glucan, which comprises linear homopolysaccharides of β-(1→4) and β-(1→3) linkages and high molecular weight viscous polysaccharides [6]. Barley β-glucan is a soluble dietary fiber that is fermented in human and animal colon to produce short-chain fatty acids (SCFAs) [7,8,9]. Thus, the fermentability of β-glucans modifies the balance and diversity of the microbiota. These prebiotic actions may be the main mechanism for the beneficial effects of barley intake. However, the mechanism for the observed improvement of glucose tolerance following barley intake remains controversial.

In a previous report, we showed that high β-glucan barley (HGB) reduced postprandial glucose rise in healthy subjects [10,11], serum cholesterol levels in hypercholesterolemic subjects [12], and abdominal fat area in moderately obese subjects [13], and regulated the calorie intake and satiety index of healthy subjects [14]. It is speculated these beneficial effects were caused by either the viscosity of β-glucan or prebiotic effects such as SCFA production.

With regard to the improvement of glucose tolerance, barley intake is reported to reduce postprandial glucose rise and the second meal effect [15]. Moreover, it is suggested that the second meal effect is the result of colonic fermentation [16]. The second meal effect [17,18] refers to the ability of foods to reduce postprandial glucose rise not only after the first meal but also after the next meal of the day. It is reported that boiled barley kernels as an evening meal decreased incremental blood glucose area at the following breakfast and increased plasma glucagon-like peptide-1 (GLP-1) at fasting and during the day by comparison with the consumption of white wheat bread [15].

G protein-coupled receptors, which sense SCFAs, are thought to contribute to the regulation of glucose metabolism [19]. Indeed, rats fed a high fiber diet had a higher plasma GLP-1 level after oral glucose administration than those fed a low fiber diet. GLP-1 is secreted from L cells, which are predominantly located in the ileum and colon [20,21]. A previous study involving rats reported that fermentable dietary fibers increase the contents of the distal digestive tract, augment GLP-1 secretion, and boost proglucagon mRNA expression [22,23,24].

Our previous report showed that barley intake increased the biosynthesis of short-chain fatty acids (SCFAs) by the gut microbiota, but we did not measure gut hormone levels [25]. Given that high β-glucan barley stimulates SCFA production, we hypothesized that a high β-glucan barley diet might result in greater GLP-1 secretion by comparison to a cellulose based diet. A recent study showed that the secretion of GLP-1 increased in mice fed high β-glucan barley and improved insulin sensitivity through modification to the gut microbiota and elevated levels of SCFAs [26]. However, another report showed that barley β-glucan promoted fermentation in cecum but did not alter glucose tolerance or insulin secretion [27].

The main aim of this study was to examine the mechanism for improvement of glucose tolerance by increases in GLP-1 secretion in mice given a diet rich in β-glucan barley. We quantified the number of L cells, mRNA expression related to L cell functions in the ileum, and GLP-1 levels in the portal vein and cecum contents of mice fed a fatty diet with and without supplementation of high β-glucan barley. An improvement of glucose tolerance mediated by GLP-1 secretion in mice fed a diet of high β-glucan barley might provide useful information for a therapeutic approach to diabetes and obesity.

## 2. Materials and Methods

### 2.1. Chemical Composition of High β-Glucan Barley

These experiments were performed using a new two-rowed, waxy, hull-less high β-glucan barley cultivar Kirari-mochi. HGB flour pearled to 60% yield was obtained from NARO (Tsukuba, Japan). Total dietary fiber was quantified using a previously published protocol [28]. The β-glucan content was determined by the McCleary procedure [29]. Protein and lipid content in HGB were analyzed by the Kjeldahl and acid hydrolysis method, respectively. The overall nutritional make-up of HGB is summarized in Table 1.

### 2.2. Experimental Design

Male C57BL/6J mice at 5 weeks of age were obtained from Charles River Laboratories Japan, Inc. (Yokohama, Japan). The mice were kept in the same holding room under a 12 h light/12 h dark regime with continual air exchange at a temperature of 22 °C (±1 °C) and humidity of 50% (± 5%). The mice were given commercial chow (NMF; Oriental Yeast Co., Ltd., Shiga, Japan) for 1 week before being divided into 2 groups matched for body mass (*n* = 8 per group). Each mouse was individually held in a plastic cage and fed the experimental diet (Table 2). The modified fatty AIN-93G diet (fat energy ratio 50%) was prepared by adding lard. Cellulose and HGB flour were included in the control and HGB diets, respectively, to give 5% total dietary fiber (Table 2). Mice were allowed ad libitum access to water and food throughout the study (83 days). Animal experiments were performed twice; the first experiment analyzed cecal GLP-1 pool size and the second experiment (*n* = 8 for the new control and experimental groups) evaluated cecal short-chain fatty acid (SCFA) pool size. Both food intake and body weight for each mouse were recorded 3 times per week. Body weight measurements were always performed at the same time of the day. On the final week, an oral glucose tolerance test (OGTT) was carried out. Mice were fasted for 6 h and then glucose (1.5 g/kg body) was orally administered. Blood glucose concentrations were analyzed from the tail using a blood glucose meter (Sanwa Kagaku Kenkyusho Co., Ltd., Aichi, Japan) at 5 timepoints (0, 15, 30, 60, and 120 min). At the end of the study, the mice were sacrificed by isoflurane/CO_2_ anesthesia after fasting for 6 h. Blood was withdrawn from portal vein (the first experiment only) and the heart and, following centrifugation, the resultant serum was stored at −80 °C. Liver, cecum, and adipose tissues were weighed. Samples of ileum were quickly saturated in RNAprotect Tissue Reagent (Qiagen, Hilden, Germany) to facilitate stabilization of RNA. The samples of cecum were stored at −40 °C until further analysis. All animal experiments were conducted with the approval of the Animal Research Committee of Otsuma Women’s University (Tokyo, Japan) (no. 12001, 16006).

### 2.3. Biochemical Analyses of the Serum

Serum cholesterol (TC), triglyceride (TG), and non-esterified fatty acids (NEFAs) were determined using a Hitachi 7180 automatic analyzer (Hitachi Ltd., Tokyo, Japan). Enzyme-linked immunosorbent assays (ELISAs) were performed to quantify serum insulin and leptin levels. GLP-1 levels in plasma derived from the portal vein were determined. Dipeptidyl peptidase IV inhibitor (Millipore, Billerica, MA, USA) was immediately added to the blood to prevent degradation of GLP-1. Quantification of GLP-1 was performed using a commercial ELISA kit (GLP-1 Active; Shibayagi Corp., Gunma, Japan).

### 2.4. Analysis of Short-Chain Fatty Acids in Cecal Digesta

Cecal SCFA content was measured using a previously described method [30]. A 7890B GC system (Agilent, Tokyo, Japan) equipped with a 5977A MSD (Agilent) was used for analysis of SCFAs. A DB-5MS capillary column (30 m × 0.53 mm) (Agilent) was used to separate the SCFAs. SCFA concentrations (expressed as μmol/cecum) were calculated by comparing their peak areas with an internal standard (crotonic acid).

### 2.5. Measurement of Total GLP-1 Level in the Cecum

Cecal tissues were extracted using Kenny’s method [31]. In brief, frozen tissue samples were extracted using an acidic ethanol solution (5 mL/g wet weight tissue). The tissue samples were subsequently homogenized and then incubated for 24 h prior to clarification by centrifuging. All extraction procedures were performed at 4 °C. The supernatant was decanted, and total GLP-1 level was measured using a total GLP-1 ELISA kit (FUJIFILM Wako Pure Chemical Corporation, Osaka, Japan).

### 2.6. mRNA Expression Analysis in the Ileum

Oligonucleotide primer sequences are shown in Table S1. mRNA levels were quantified by real-time PCR (QuantStudio3 RT-PCR system; Applied Biosystems, Foster City, CA, USA). Complementary DNA was used to quantify the mRNA expression levels according to the 2^−ΔΔCT^ method with a commercial PCR Master Mix (Thermo Fisher Scientific, Waltham, MA, USA). Data were assessed from threshold cycle (Ct) values, which indicate the cycle number at which the fluorescence signal reaches a fixed threshold that is well above background. ΔCT is the difference in Ct values for genes of interest compared to transcription factor II B (TFII B), which served as a reference. The ΔΔCT is the difference between the ΔCT for the control group and the ΔCT for the HGB group. Relative expression levels are shown as fold differences compared to the control group (arbitrary units).

### 2.7. Measurement of the Number of L Cells in the Ileum

After collecting the ileum, samples were immersed in 4% paraformaldehyde/phosphate buffer and fixed, then dehydrated, degreased, paraffin-penetrated, paraffin-embedded, sliced, spread, and dried at the Institute of Nutrition and Pathology, Inc. (Kyoto, Japan). Deparaffinization and rehydration were performed with xylene, anhydrous ethanol, 99% ethanol, and 70% ethanol. Endogenous peroxidase inhibition was achieved with 30% (*v/v*) H_2_O_2_ and methanol. Then, each ileal section was blocked using diluted 5% (*v/v*) goat serum (Normal) (DakoCytomation, Glostrup, Denmark) and 0.5% (*w/v*) BSA/PBS (bovine serum albumin/phosphate buffered saline). Anti-GLP-1 (7–36) -NH2; rabbit Ab (Yanaihara Institute Inc., Shizuoka, Japan) was used as the primary antibody. For the secondary antibody, Histofine Simple Stain Mouse MAX-PO (Rabbit) (Nichirei Bioscience Inc., Tokyo, Japan) with 1% (*v/v*) mouse serum (Normal) (DakoCytomation, Glostrup, Denmark) was used. After the antigen–antibody reaction, samples were colored with the ENVISION kit/HRP (horseradish peroxidase) (DAB; 3,3’-Diaminobenzidine) (DakoCytomation, Glostrup, Denmark). In addition, nuclear staining was performed with hematoxylin and the samples were enclosed with Mount Quick (Daido Sangyo Corporation, Saitama, Japan). Each ileal section was photographed under a light microscope. From images of the ileum, we measured the number of cells per unit area (1 mm^2^) using WinROOF (Version 6.0.1; Mitani Corporation, Fukui, Japan).

### 2.8. Statistical Analyses

Data are given as the mean ± standard error of the mean (SE). Comparisons of data between two groups were performed by unpaired Student’s *t*-test (JMP Version 14.2.0; SAS Institute Inc., Cary, NC, USA). Time-dependent changes in the blood glucose levels during the OGTT were analyzed by two-way ANOVA (diet × time). A *p*-value of <0.05 was considered statistically significant.

## 3. Results

### 3.1. Gross Changes to Mice during the Study Period

No significant differences were identified between the two experimental groups in terms of weight gain, food intake, and food efficiency ratio (Table 3). Similar results were obtained for the second experiment (Table S2). Organ weights are shown in Table 4. There were no significant differences in liver weight and the weights of retroperitoneal, epididymal, and mesenteric fat between the two experimental groups. However, the weight of cecum with digesta was significantly higher in the HGB group compared to the control group. Similar results were obtained for the second experiment (Table S3).

### 3.2. Biochemical Markers in the Serum

The concentrations of serum biochemical markers are shown in Table 5. There were no significant differences in serum total cholesterol, triglyceride, NEFA, glucose, insulin, and leptin concentrations between the two experimental groups.

### 3.3. Assessment of Glucose Tolerance

**Figure 1:**
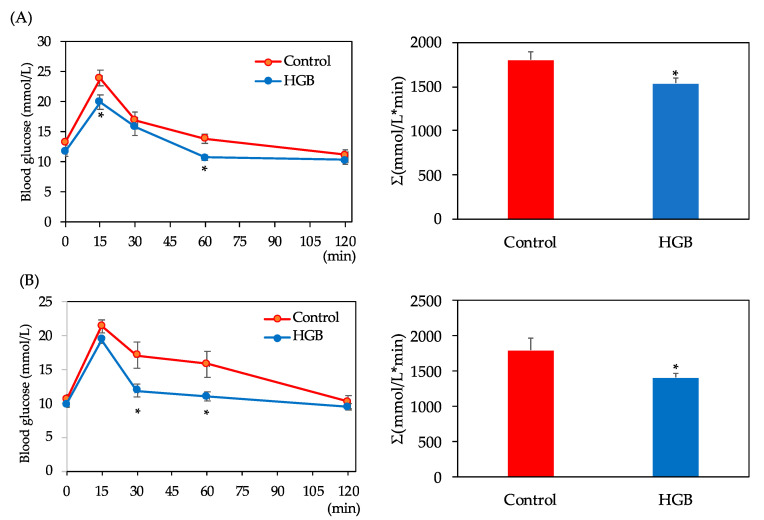
Blood glucose levels in the oral glucose tolerance test (OGTT) and area under the curve of blood glucose for (**A**) the first experiment for cecal glucagon-like peptide-1 (GLP-1) analysis and (**B**) the second experiment for cecal short-chain fatty acid (SCFA) analysis. Data are shown as mean ± standard error of the mean (SE). Means marked by an asterisk differ significantly (Student’s *t*-test, * *p* < 0.05). HGB; high β-glucan barley.

The OGTT and area under the curve of blood glucose (AUC) results are shown in Figure 1. Significant interaction (diet × time) in the blood glucose levels was not observed, but blood glucose levels in the HGB group were significantly lower than in the control group (main effect was significant), as shown by two-way ANOVA. Blood glucose levels after glucose administration were significantly lower in the HGB group compared with the control group at 15 and 60 min in the first experiment. In the second experiment, significant differences between the control and HGB groups were also observed at 30 and 60 min. AUC was significantly lower in the HGB group compared with the control group in both experiments.

### 3.4. Analysis of Short-Chain Fatty Acids in Cecal Digesta

**Figure 2:**
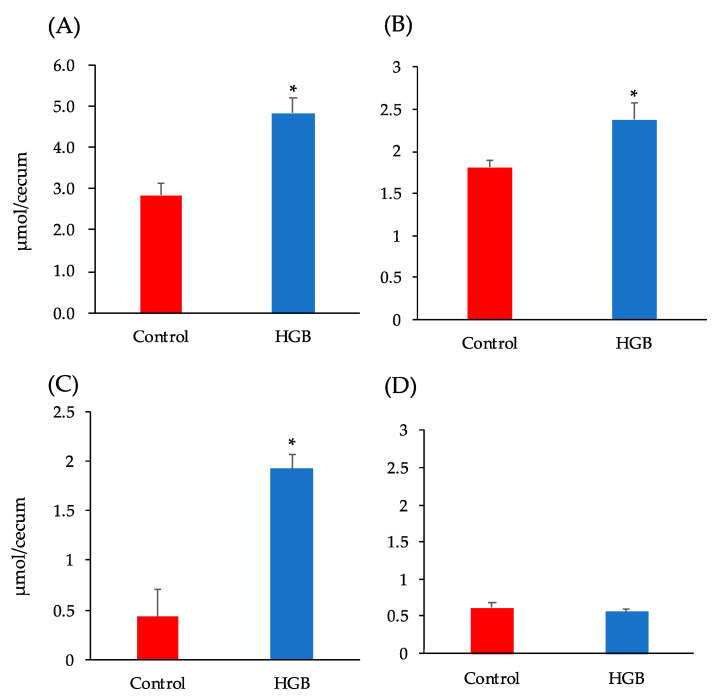
Short-chain fatty acid (SCFA) content in the cecal digesta of mice fed the test diets. (**A**) Total SCFA, (**B**) Acetate, (**C**) Propionate, (**D**) Other SCFA (the sum of the concentrations of formate, *n*-butyrate, *iso*-butyrate, *iso*-valerate, and valerate). Bars represent means and standard error of the mean (SE), *n* = 8. Means marked by an asterisk differ significantly (Student’s *t*-test, * *p* < 0.05). HGB; high β-glucan barley.

The cecal pool size of SCFAs is shown in Figure 2. Total SCFA concentrations, as well as acetate and propionate concentrations, were significantly higher in the HGB group than the control group.

### 3.5. Concentrations of GLP-1 in the Portal Vein and Cecum

The concentration of GLP-1 in the portal vein and cecum are shown in Table 6. Portal vein and cecal GLP-1 levels were significantly higher in the HGB group compared with the control group.

### 3.6. mRNA Expression Levels in the Ileum Determined by q-PCR

**Figure 3:**
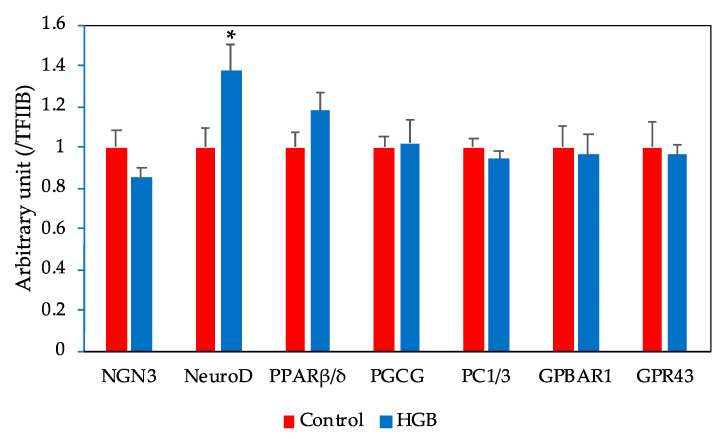
Expression of mRNAs related to L cell function in the ileum bars represent means and standard error of the mean (SE), *n* = 8. Means marked with an asterisk differ significantly (Student’s *t*-test, * *p* < 0.05). NGN3, neurogenin 3; NeuroD, neurogenic differentiation factor; PPARβ/δ, peroxisome proliferator-activated receptor β/δ; PGCG, proglucagon; PC1/3, prohormone convertase 1/3; GPBAR1, G-protein-coupled bile acid receptor 1; GPR43, G-protein-coupled receptor 43; TFIIB, transcription factor II B. HGB; high β-glucan barley.

The experimentally determined mRNA levels in the ileum are given in Figure 3. No significant differences were found in peroxisome proliferator-activated receptor β/δ (PPARβ/δ), proglucagon (PGCG), prohormone convertase 1/3 (PC1/3), G-protein-coupled bile acid receptor 1 (GPBAR1), and G-protein-coupled receptor 43 (GPR43) between the two experimental groups. Likewise, there were no significant differences in neurogenin 3 (NGN3). By contrast, the mRNA expression level of NeuroD was elevated in the HGB group in comparison with the control group.

### 3.7. Number of L Cells in the Ileum

**Figure 4:**
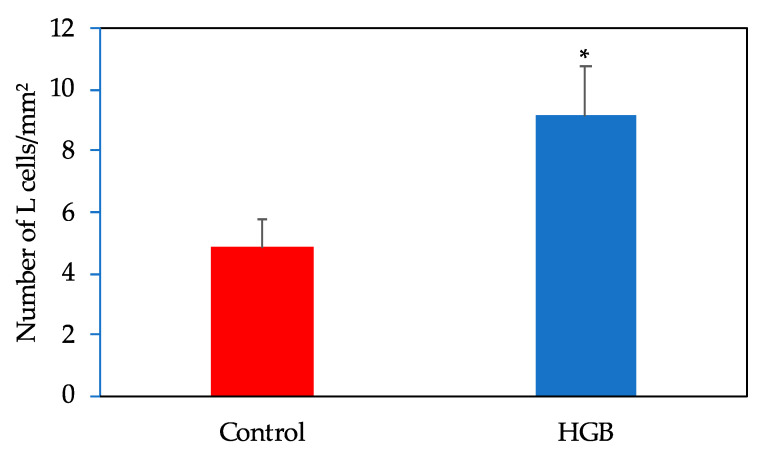
Number of L cells in the ileum. Data are shown as mean ± standard error of the mean (SE). * Significantly different from the control group (*p* < 0.05). HGB; high β-glucan barley.

A representative image of GLP-1 positively stained cells of the ileum is shown in Supplementary Figure S1. The number of L cells in the ileum are shown in Figure 4. L cell numbers were significantly higher in the HGB group compared with the control group.

## 4. Discussion

Herein, we investigated the mechanism for improvement of glucose tolerance in mice fed a high-fat diet containing HGB. Results from this study indicated that HGB intake improved glucose tolerance in OGTT. GLP-1 levels in the portal vein and cecal pool size were significantly increased in the HGB group. These findings suggest that the effect of HGB on glucose metabolism was mediated by increased GLP-1 secretion. Previous studies in human [15] and animal experiments [26] showed that the serum GLP-1 concentrations were increased by the barley intake. However, effects of HGB intake on the L cell function related to the GLP-1 secretion were not observed in the previous experiments. This is the first evidence to show an increase in the number of ileal L cells causing an increase in intestinal GLP-1 pool size and GLP-1 secretion without the alteration of mRNA expression related to GLP-1 secretion.

These results, together with those from our previous report [25], suggest that augmentation in the number of L cells and the subsequent elevation in the level of GLP-1 were induced by production of SCFAs. Thus, a new mechanism for increasing GLP-1 secretion by HGB intake is proposed. It has been generally recognized that the preventive effect of barley intake on postprandial blood glucose rise is triggered by delayed gastric emptying time due to the viscous properties of barley β-glucan and the suppression of digestion and absorption of starch in barley [32,33]. However, our previous report showed that improvement of glucose tolerance became more pronounced when HGB with partially hydrolyzed β-glucan was supplemented into the test diet [34]. It is possible that a prebiotic effect, such as intestinal fermentation, contributed more to the improved glucose tolerance than the physicochemical effect, such as increased viscosity. Furthermore, HGB imparts a second meal effect, which is induced through intestinal fermentation [15]. In a human intervention study, it was reported that the postprandial blood glucose rise was suppressed when a standard diet without barley was consumed at lunch after the intake of a HGB diet at breakfast [35,36].

Our experiments showed no difference in serum insulin concentration between the two groups, but the active GLP-1 level in the portal vein of the HGB group was significantly elevated by comparison to the control group. The total GLP-1 pool size of the cecal contents in the HGB group was also significantly elevated compared to the control group. GLP-1 is produced by prohormone convertase 1/3 from the precursor proglucagon in L cells. However, changes in the expression levels of peroxisome proliferator-activated receptor β/δ (PPARβ/δ), proglucagon, and prohormone convertase 1/3 related to GLP-1 secretion in the ileum were not observed. The expression levels of NeuroD, which is thought to reflect the number of L cells, increased significantly in the HGB group. These results indicated that the improvement of glucose tolerance following HGB intake is fostered by an increase in GLP-1 secretion accompanying the increased number of L cells.

Several studies propose secretion of GLP-1 and gastric inhibitory peptide (GIP) may be coupled with changes to the microflora of the lower gut as well as glucose metabolism [37,38,39,40]. In 1996, the first published report appeared, suggesting fermentation in the lower digestive tract promoted an increase of GLP-1 secretion [22]. It was also reported that a prebiotic oligofructose led to an increase in total cecal GLP-1 concentration [41]. Similar effects were observed with resistant starch, which increased the concentration of plasma GLP-1 [42,43]. Our study indicated prebiotic barley β-glucan is one of the dietary fibers that promotes GLP-1 secretion.

Previous studies report that inert dietary fibers did not enhance colonic proliferation of epithelial cells, although fermentable soluble fibers were able to promote proliferation in the lower gut, which is associated with increased enteroglucagon secretion [44,45]. Enteroglucagon, which regulates intestinal epithelial cell proliferation and acts as a trophic factor for the intestinal mucosa, is synthesized in the L cells of the ileum and colon. It is speculated that increases in the number of L cells is mediated by the trophic action of enteroglucagon. Luminal infusion of acetate and butyrate was reported to significantly increase colonic GLP-1 secretion, whereas propionate had no such effect [46]. Moreover, SCFAs may increase the release of GLP-1 via the SCFA receptor GPR43, which is expressed on the L cells [47].

A major limitation of this study was the lack of data regarding serum insulin and GLP-1 concentrations during the OGTT. Future studies are needed to further elucidate the relationship between GLP-1 release and insulin response by HGB intake.

## 5. Conclusions

In conclusion, we confirm that the observed improvement of glucose tolerance by HGB intake was induced by an increase in L cell number and the subsequent enhancement in GLP-1 secretion. We demonstrated for the first time a relationship between the level of GLP-1, the number of L cells, and the level of NeuroD mRNA in the lower digestive tract. On the basis of these findings, we propose that the presence of barley β-glucan in the lower gut increases L cell differentiation by upregulation of NeuroD gene expression. The present results are consistent with the notion that events occurring in the lower gut, such as fermentation and alteration of gut microbiota, exert a key mechanism on glucose metabolism.

## Figures and Tables

**Table 1:** Nutritional components of high β-glucan barley (HGB).

	(g/100 g)
Moisture	9.1
Fat	1.8
Protein	12.8
Ash	0.7
Available carbohydrate	65.7
Total dietary fiber	9.9
β-glucan	5.4

Available carbohydrate: (100 − (“Moisture” + ”Fat” + ”Protein” + ”Ash” + ”Total dietary fiber”)).

**Table 2:** Composition of the experimental diets.

		(g/kg Diet)
	Control	HGB
Casein	200	120.6
L-cystine	3	3
Corn starch	197.486	-
Dextrinized corn starch	132	-
Sucrose	100	62.886
Soybean oil	70	70
Lard	200	190.9
Cellulose	50	-
HGB flour	-	505.1 *
AIN-93G mineral mixture	35	35
AIN-93 vitamin mixture	10	10
Choline bitartrate	2.5	2.5
*t*-Butylhydroquinone	0.014	0.014

* β-glucan content in the HGB diet was 27.3 g/kg diet. Total energy in the control and HGB diets were 4.96 kcal/g diet: carbohydrate 35 en %, protein 14 en %, fat 50 en %. HGB; high β-glucan barley.

**Table 3:** Weight gain, food intake, and food efficiency ratio.

	Control	HGB
Initial weight (g)	18.5 ± 0.2	18.5 ± 0.2
Final weight (g)	41.2 ± 0.7	42.4 ± 0.9
Body weight gain (g/d)	0.27 ± 0.01	0.30 ± 0.01
Food intake (g/d)	2.8 ± 0.0	3.0 ± 0.1
Food efficiency ratio (%)	9.73 ± 0.41	9.92 ± 1.06

Values are means ± standard error of the mean (SE), *n* = 8. HGB; high β-glucan barley.

**Table 4:** Weight of organs.

	Control	HGB
Liver (g)	1.47 ± 0.06	1.56 ± 0.09
Cecum with digesta (g)	0.28 ± 0.02	0.38 ± 0.02 *
Total abdominal fat	4.53 ± 0.20	4.38 ± 0.16
Retroperitoneal fat (g)	0.95 ± 0.05	0.91 ± 0.02
Epididymal fat (g)	2.61 ± 0.11	2.48 ± 0.01
Mesenteric fat (g)	0.97 ± 0.10	0.99 ± 0.10

Values are means ± standard error of the mean (SE), *n* = 8. Asterisk indicates a significant difference (Student’s *t*-test, * *p* < 0.05). HGB; high β-glucan barley.

**Table 5:** Serum biochemical concentrations.

	Control	HGB
Cholesterol (mmol/L)	5.60 ± 0.34	5.96 ± 0.55
Triglyceride (mmol/L)	0.84 ± 0.11	0.67 ± 0.04
NEFA (μmol/L)	639.8 ± 45.5	728.0 ± 33.0
Glucose (mmol/L)	16.61 ± 0.86	14.88 ± 0.55
Insulin (ng/mL)	6.10 ± 1.03	8.36 ± 1.22
Leptin (ng/mL)	71.35 ± 7.22	73.48 ± 6.14

Values are means ± standard error of the mean (SE), *n* = 8. NEFA; non-esterified fatty acids. HGB; high β-glucan barley.

**Table 6:** Portal vein and cecal GLP-1 levels.

	Control	HGB
Portal vein active GLP-1 (7-36) levels (pg/mL)	50.0 ± 9.5	81.4 ± 10.0 *
Cecal total GLP-1 pool size (ng/cecum)	31.1 ± 7.4	64.5 ± 8.4 *

Values are means ± standard error of the mean (SE), *n* = 8. Means marked with an asterisk differ significantly (Student’s *t*-test, * *p* < 0.05). HGB; high β-glucan barley.

## Data Availability

Data are contained within the article or supplementary material.

## Supplementary Materials

The following are available online at https://www.mdpi.com/2072-6643/13/2/527/s1: Table S1: Primers used for real-time reverse transcription polymerase chain reaction. Table S2: Body weight gain, food intake, and food efficiency ratio (2nd Exp). Table S3: Weight of organs (2nd Exp). Supplementary Figure S1: Representative GLP-1 staining of cells from the ileum: (a) control and (b) HGB staining of GLP-1-positive cells.
