# Supplementary material for: High β-Glucan Barley Supplementation Improves Glucose Tolerance by Increasing GLP-1 Secretion in Diet-Induced Obesity Mice
Source: Nutrients. 2021 Feb 6;13(2):527. doi: 10.3390/nu13020527 (PMC7915888; doi:10.3390/nu13020527)
Supplement: Supplementary file 1 [file nutrients-13-00527-s001.pdf]

Supplementary Table S1 Primers used for the real-time reverse transcription polymerase chain reactions.

	Forward	Reverse
Ileal L cell function		
NeuroD	5'-CTTGGCCAAGAACTACATCTGG-3'	5'-CGTGTTTGAAAGAGAAGTTGCC-3'
NGN3	5'-AAGAGCGAGTTGGCACTCAGC-3'	5'-AAGCTGTGGTCCGCTATGCG -3'
PPARβ/δ	5'-ACTTGGCGTGGCGCCTGC-3'	5'-AGCGGTGTGGGTATGCGCA-3'
PGCG	5'-ATTGCCAAACGTCATGATGA-3'	5'-GGCGACTTCTTCTGGGAAGT-3'
PC1/3	5'-AGACAGCATTACACCATCTCTA-3'	5'-AGAACACTTCTCTGCATACCAAGGT-3'
GPBAR1	5'-AACGCTACATGGCAGTGTTG-3'	5'-GGAGGCCATAAACTTCCAGGTAGA-3'
GPR43	5'-GGGATCTGGGTCACATGCTTAT-3'	5'-ATGTCAGACAGACGGGTACCAA-3'
Reference		
TFIIB	5'-ACCAGCCGTTTGGATGCTC-3'	5'-CCCACATCAATAACTCGGTC-3'

NeuroD, neurogenic differentiation factor; NGN3, neurogenin 3; PPARβ/δ, peroxisome proliferator-activated receptor β/δ; PGCG, proglucagon, PC1/3, prohormone convertase 1/3; GPBAR1, G-protein–coupled bile acid receptor 1; GPR43, G-protein-coupled receptor 43; TFIIB, transcription factor II B.

Supplementary Table S2 Body weight gain, food intake, and food efficiency ratio (2nd Exp)

	Control	HGB
Initial weight (g)	20.1±0.3	20.1±0.3
Final weight (g)	41.3±1.5	43.6±0.8
Body weight gain (g/d)	0.20±0.01	0.22±0.01
Food intake (g/d)	2.89±0.04	2.89±0.05
Food efficiency ratio (%)	6.78±0.37	7.51±0.18

Values are means ±standard error of the mean (SE), n=8. HGB; high β-glucan barley.

Supplementary Table S3 Weight of organs (2nd Exp)

	Control	HGB
Liver（g）	1.59±0.17	1.54±0.12
Cecum with digesta (g)	0.22±0.01	0.26±0.01*
Retroperitoneal fat（g）	0.93±0.07	1.08±0.04
Epididymal fat（g）	2.11±0.14	2.38±0.15
Mesenteric fat（g）	0.95±0.18	1.20±0.09

Values are means ±standard error of the mean (SE), n=8.

Means with suffixed superscript letters differ significantly (Student's t-test, *$p < 0.05$ vs. control). HGB; high β-glucan barley.

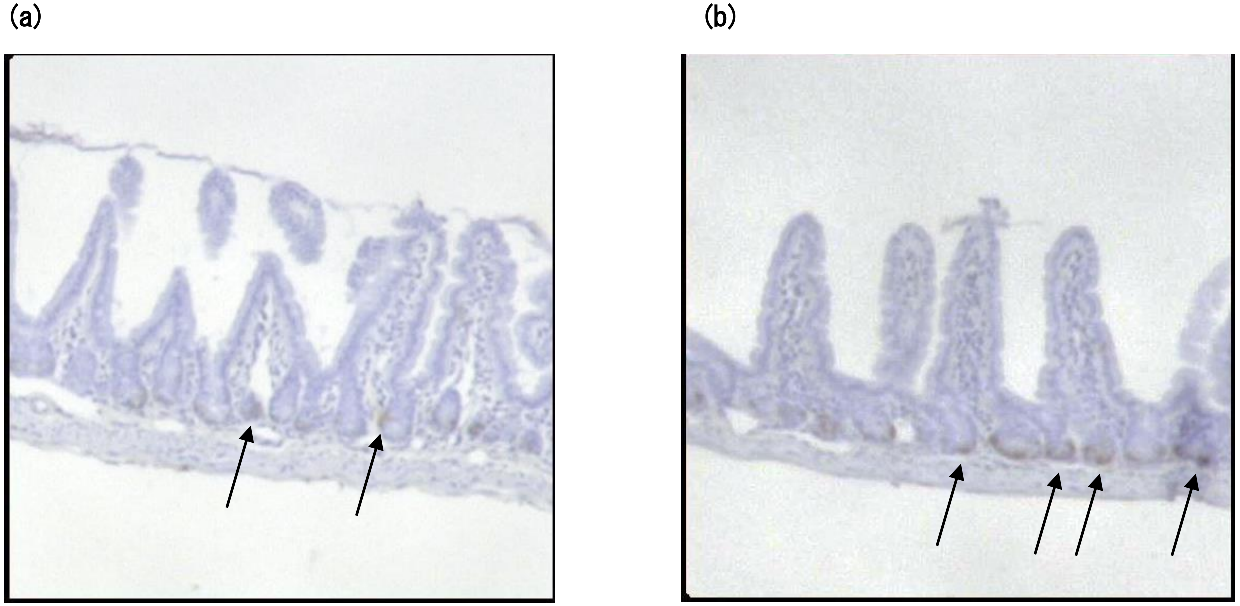

Supplementary Figure S1. Representative GLP-1 staining of cells from the ileum. (a) Control, (b) HGB, staining of GLP-1 positive cells (highlighted by arrows).× 200 magnification (one side length; 500μm)

HGB; high β-glucan barley.
